# Ticks: More Than Just a Pathogen Delivery Service

**DOI:** 10.3389/fcimb.2021.739419

**Published:** 2021-09-01

**Authors:** Jason M. Park, Adela S. Oliva Chávez, Dana K. Shaw

**Affiliations:** ^1^Program in Vector-Borne Disease, Department of Veterinary Microbiology and Pathology, Washington State University, Pullman, WA, United States; ^2^Department of Entomology, Texas A&M University, College Station, TX, United States

**Keywords:** tick-borne diseases, vector competence, tick-borne pathogens, arthropod immunity, tick saliva, salivary exosomes

## Introduction

Ticks are important pathogen vectors that are a growing threat owing to expanding habitats and longer active periods (Dennis et al., 1998; Ostfeld and Brunner, 2015; Beard et al., 2016; Eisen et al., 2016a; Eisen et al., 2016b). The incidence of reported tick-borne diseases in the United States has risen significantly over the last decade with cases more than doubling (Rosenberg, 2018). Various issues hinder the ability to combat tick-borne disease including the lack of available vaccines, misdiagnosis and the emergence of antibiotic/antiparasitic resistance (Masters et al., 1998; Aguero-Rosenfeld et al., 2005; Wormser et al., 2006; Stanek et al., 2012; Molins et al., 2017). Much of what is known about tick-borne pathogens is centered around interactions with mammalian hosts, as this is where pathological outcomes are often observed. Comparatively less is understood about pathogen interactions with the arthropod vector.

**Figure 1:**
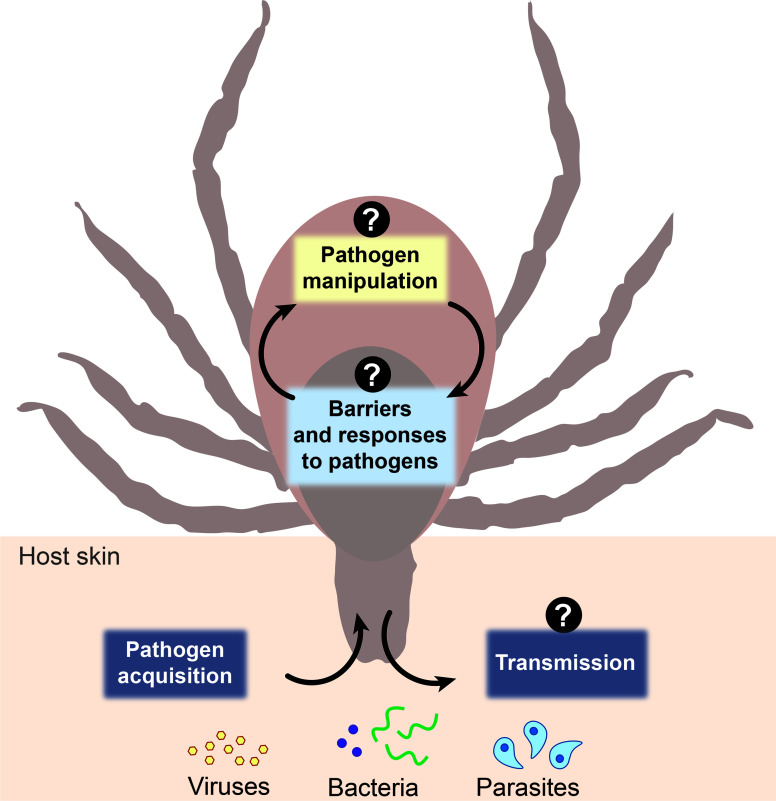
Schematic representation of three aspects of tick-microbe interactions. The vector-side of tick-borne disease is open for discovery. Question marks represent topics in the field that remain incompletely understood including arthropod barriers to colonization and responses to pathogens, how microbes manipulate the arthropod to colonize and survive within the tick, and what the role and composition of salivary secretions are during pathogen transmission dynamics.

The environmental milieu between vertebrate hosts and arthropods vectors differs significantly with disparities in body temperature, physiological architecture, immunological potential, and nutrient availability. Ticks play a crucial role in not only harboring pathogens, but also priming them for transmission (De Silva and Fikrig, 1995; Ramamoorthi et al., 2005; Fikrig and Narasimhan, 2006; Dunham-Ems et al., 2009; Shaw et al., 2016; Chávez et al., 2019; Oliva Chávez et al., 2021). Herein, we will discuss three aspects of tick-microbe interactions: barriers to colonization, microbial manipulation, and saliva-transmission dynamics. We contend that, rather than a passive vessel, the arthropod is a dynamic environment that shapes pathogen adaptation, selection, and transmission (**Figure 1**). To advance the field of tick-borne disease, cross-disciplinary approaches are needed to uncover fundamental basics centered on the vector-side of this equation, which may pave the way for novel disease prevention strategies.

## Colonization: Overcoming Barriers

There are several barriers that must be overcome for a microbe to successfully colonize the tick. The peritrophic membrane and midgut epithelium are physical barriers that pathogens first encounter when colonizing a vector with an incoming bloodmeal. Different microbes occupy specific niches, but all must eventually cross the midgut and migrate to the salivary glands for transmission to a host. For example, *Borrelia burgdorferi* will cross the peritrophic membrane and colonize the midgut epithelium where it will remain during the molt, until the tick feeds again (Narasimhan et al., 2014; Narasimhan et al., 2017). In contrast, *Anaplasma phagocytophilum* rapidly escapes the midgut and colonizes the salivary glands (Hodzic et al., 1998; Liu et al., 2011; Abraham et al., 2017). Strategies used by microbes to respond to stimuli and traverse physical barriers at specific times are not fully understood (Sonenshine and Macaluso, 2017).

A secondary barrier to colonization is the tick immune system (Oliva Chávez et al., 2017; Fogaça et al., 2021). Some immune pathways are described as restricting vector-borne microbes, such as the Immune Deficiency pathway (Rosa et al., 2016; Capelli-Peixoto et al., 2017; Shaw et al., 2017; Carroll et al., 2019), the JAK-STAT pathway (Liu et al., 2012; Smith et al., 2016), the RNAi pathway (Rückert et al., 2014; Schnettler et al., 2014; Grubaugh et al., 2016; Hart and Thangamani, 2021), and phagocytic hemocytes (Coleman et al., 1997; Dunham-Ems et al., 2009; Talactac et al., 2021); however, vectored pathogens are still able to colonize the tick. Whether this is through microbial-mediated immune evasion/suppression or through immunological tolerance from the arthropod is not clearly defined (Sonenshine and Macaluso, 2017; Shaw et al., 2018; Boulanger and Wikel, 2021; Rosche et al., 2021). Prolonged nutrient limitation between bloodmeals and competition with the resident microbiota/endogenous virome are also restrictive forces that must be dealt with (Bell-Sakyi and Attoui, 2013; Shaw et al., 2018; O’Neal et al., 2020; Samaddar et al., 2020; Bonnet and Pollet, 2021; Narasimhan et al., 2021). To what extent these factors limit colonization and/or survival is not clear, but are undoubtedly pressures that must be coped with by the pathogen.

A comprehensive understanding on this topic is limited by the scope of basic tick biology knowledge. For example, although important morphological and structural descriptions of tick hemocytes have been reported (Dolp, 1970; Brinton and Burgdorfer, 1971; Dolp and Hamdy, 1971; Pereira et al., 2001; Kadota et al., 2003; Matsuo et al., 2004; Borovicková and Hypsa, 2005; Kotsyfakis et al., 2015), the categorization of specific subpopulations and how they function during infection remains undefined. Modern technical advancements may provide insight into the molecular and cellular biology of discrete hemocyte subsets and how they may impact vector competence. Historically, much of what is known about arthropods is informed by the insect model organism *Drosophila*. This has been and continues to be a powerful system for elucidating important biological concepts, but there are significant physiological and genetic differences between insects and ticks which diverged approximately 450 million years ago (Mans et al., 2016; Beati and Klompen, 2019). For instance, *Ixodidae* ticks do not have a prophenoloxidase system, which is an important insect defense against infection (Zhioua et al., 1997; Sonenshine and Roe, 2014; Palmer and Jiggins, 2015; Gulia-Nuss et al., 2016). β-1,3-glucan recognition proteins are also not found in ticks, which recognize fungal organisms and induce immune responses in insects (Ferrandon et al., 2007; Bechsgaard et al., 2016). The extent of these differences as they relate to vector competence and microbial colonization remains elusive, but recent findings imply that there is much to learn (Palmer and Jiggins, 2015; Rosa et al., 2016; Capelli-Peixoto et al., 2017; Shaw et al., 2017; Sonenshine and Macaluso, 2017; Carroll et al., 2019). These studies highlight the importance of examining concepts directly in ticks to elucidate what barriers are unique to these non-model organisms and how transmissible microbes are manipulating them.

## Microbial Hacking: Manipulating the Arthropod

Microbial strategies to colonize the tick is an understudied area in the field, but likely involve infection determinants that are specific to the arthropod. The overarching goal is to overcome barriers within the tick to enable growth, survival, and transmission to a host. Two categories of microbial molecules that facilitate these goals are surface-localized proteins and secreted effectors. Many microbial surface proteins play essential roles during mammalian infection to enable pathogen adherence, tissue invasion, immune evasion, and dissemination (Sonenshine and Macaluso, 2017). A smaller number of proteins are known to act at the vector-pathogen interface, but those that have been identified perform critical tick-specific functions. For example, *B. burgdorferi* OspA binds to Tick Receptor for OspA (TROSPA) to facilitate midgut colonization and persistence through the molt (Pal et al., 2004). Post translational modifications of surface proteins can also be critical for survival, as is the case with the *A. phagocytophilum* O-methyltransferase which modifies Msp4 and is essential for growth in tick cells (Oliva Chávez et al., 2015). The full repertoire of surface proteins used by tick-borne microbes to manipulate interactions with the vector remains largely undefined.

Multiple tick-borne pathogens employ specialized secretion apparatuses to deliver effector molecules. One example is the Type IV Secretion System (T4SS) used by Rickettsial pathogens, including *Anaplasma* spp., *Rickettsia* spp. and *Ehrlichia* spp. (Gillespie et al., 2010; Rikihisa, 2017). During mammalian infections, T4SS-injected effectors manipulate signaling cascades, alter gene transcription, evade cellular defenses, and liberate essential nutrients. For instance, *Rickettsia* effector Risk1 promotes internalization, phagosomal escape, and autophagosomal escape by modifying phosphoinositides (Voss et al., 2020). *A. phagocytophilum* and *E. chaffeensis* effectors Ats1 and Etf1 inhibit host apoptosis and stimulate autophagy to liberate nutrients (Niu et al., 2010; Lin et al., 2016). Most secreted effectors encoded by Rickettsial pathogens are uncharacterized, with only a few that have known functions in mammals and even less with identified roles in the tick.

Although little is known about infection determinants for colonizing the arthropod, evidence suggests that there are protein expression patterns specific for the tick. The cattle pathogen, *Anaplasma marginale*, differentially transcribes 34 surface protein-encoding genes during growth in tick cells relative to mammalian blood (Hammac et al., 2014), indicating there are arthropod-specific surface modifications yet to be dissected. Secreted effectors can have species-specific roles, as is the case for the non-tick-borne pathogen *Legionella pneumophila* (Park et al., 2020). Machine learning algorithms predict that *A. phagocytophilum* encodes up to 48 T4SS-secreted effectors, some of which are differentially expressed during growth within mammalian *versus* tick cells (Nelson et al., 2008; Esna Ashari et al., 2019). This suggests that the effector repertoire may enable host switching between vertebrate hosts and the arthropod. Identifying and characterizing microbial infection determinants that mediate tick-pathogen interactions will not only define infection strategies used by the pathogen, but also identify pathways that are manipulated within the vector.

## Saliva-Transmission Dynamics: A Free Ride or Hijacking the Situation?

Modulation of immune responses, coagulation, and hemostasis at the bite site through the secretion of salivary molecules is a central component of hematophagy (Tirloni et al., 2020a). Likewise, vector-borne pathogens rely on these molecules for transmission and establishment of infection. Several studies have shown that vector transmission promotes pathogen infection and intensifies disease severity (Grasperge et al., 2014; Pingen et al., 2016; Onyango et al., 2020; Karim et al., 2021). This phenomenon is well-known and referred to as saliva-assisted transmission (Nuttall, 2019; Oliva Chavez, 2021). Although some tick saliva molecules that facilitate transmission have been identified, our knowledge of the cellular and molecular mechanisms involved are still underdeveloped.

Tick saliva is a complex and dynamic mix of molecules which change in response to different hosts and over the course of tick feeding (Karim and Ribeiro, 2015; Tirloni et al., 2017; Nuttall, 2019; Tirloni et al., 2020b). Non-protein salivary molecules include lipids, such as Prostaglandin E2 (PGE2) (Bowman et al., 1996), metabolites, like adenosine (Oliveira et al., 2011), and microRNAs (Hackenberg et al., 2017; Malik et al., 2019; Nawaz et al., 2020a). Protein salivary components are the best studied and defined as the “sialome” or “sialoverse” (Mans, 2020). Recently, hundreds of thousands of tick salivary gland transcripts were identified and bioinformatically classified into protein families such as lipocalins, metalloproteases, basic tail secreted proteins, etc. (Ribeiro and Mans, 2020). Relationship analyses indicate that a high rate of evolution and intragenic recombination is driving the expansion of these protein families (Ribeiro and Mans, 2020). Compared to the number of transcriptomic and proteomic studies done in salivary glands and saliva from hard ticks (*Ixodidae*), only a few soft ticks have been explored and the gene expression dynamics during feeding have only recently been described (Oleaga et al., 2021). The Argasid sialome is comprised of several protein families similar to those identified in hard ticks (Martins et al., 2021; Oleaga et al., 2021). Although many proteins appear to be conserved between tick species, functionality has not been extensively investigated and represents an important knowledge gap. Further, a significant number of expressed genes remain uncharacterized and their role in tick feeding and/or pathogen transmission is therefore unknown (Ribeiro and Mans, 2020; Martins et al., 2021; Oleaga et al., 2021).

Recently, many salivary molecules were discovered to be secreted within extracellular vesicles (Nawaz et al., 2020a; Nawaz et al., 2020b; Zhou et al., 2020; Oliva Chávez et al., 2021), which impact host immune responses, wound healing and feeding success of the tick (Karim et al., 2002; Karim et al., 2005; Alarcon-Chaidez et al., 2009; Villarreal et al., 2013; Shaw et al., 2016; Zhou et al., 2020; Oliva Chávez et al., 2021; Pham et al., 2021). Interestingly, salivary extracellular vesicles can modulate the outcome of vector-borne infections, as is the case for *A. phagocytophilum* and *Francisella tularensis* (Oliva Chávez et al., 2021). Extracellular vesicles also facilitate vector-borne virus infection and transmission (Silvas et al., 2016; Vora et al., 2018; Zhou et al., 2018; Reyes-Ruiz et al., 2019; York et al., 2021). For instance, the *Haemaphysalis longicornis*-borne virus SFTS (Severe Fever with Thrombocytopenia Syndrome) exploits host-derived vesicles to invade uninfected cells and evade antibody defenses (Silvas et al., 2016). Extracellular vesicle cargo can be altered by intracellular vector-borne pathogens, not only by adding pathogen-derived contents but also by changing the composition of host-derived molecules within the vesicles (Regev-Rudzki et al., 2013; Silvas et al., 2016; Vora et al., 2018; Jiang et al., 2020; York et al., 2021). The malaria-causing parasite *Plasmodium falciparum* commandeers red blood cells exosomes to communicate and exchange genetic material with other parasites during infection (Regev-Rudzki et al., 2013). The mite-borne rickettsia *Orientia tsutsugamushi* influences serum derived exosome contents during mammalian infection by altering the concentrations of specific miRNAs (Jiang et al., 2020). Similarly, *A. phagocytophilum* changes the contents of exosomes from infected tick cells (Oliva Chávez et al., 2021). To what extent other tick-borne pathogens alter salivary extracellular vesicles and how this may affect immunomodulatory potential and/or pathogen transmission is an area that remains ripe for discovery.

## Concluding Remarks

The distinctive biology of ticks and their long co-evolutionary relationship with the pathogens they vector presents a unique set of challenges to the field. This is partially owing to a lack of genetic tools and reagents available for non-model organisms. Progress on these fronts will benefit from creative approaches and cross-disciplinary interactions/collaborations with colleagues across fields. As noted in Sullivan, 2015, if individuals with training in classic model organisms were to “try their chops” at the genetics, cell biology and biochemistry of ticks and tick-borne microbes it may facilitate the development of novel tools that would lead to breakthroughs in the field.

Despite these unique challenges, we offer the opinion that examining the biology of ticks and interactions with the pathogens they transmit will uncover new paradigms in the arthropod-borne disease triangle that could translate to different systems (**Figure 1**). For example, microbial effectors that are expressed in the arthropod may be targeting undiscovered cellular processes that dictate vector competence, which may be conserved across arthropod vectors. Likewise, understanding the biology of ticks will yield unique insights into requirements for microbial survival, persistence, and transmission in the face of host and vector immunity. With growing accessibility to technology and improved techniques that increase the limits of detection, pulling back the curtain on non-model organism biology is becoming feasible. This information will improve our fundamental knowledge in vector biology and microbiology and may pave the way for developing innovative approaches to control tick-borne disease.

## Author Contributions

JP, AO, and DS designed and wrote this opinion. All authors contributed to the article and approved the submitted version.

## Funding

This work was supported by National Institute of Food and Agriculture (NIFA) United States Department of Agriculture Animal Health grant TEX09902 and Texas A&M University, National Institute of Health grants R21AI151412, R21AI154023, R21AI139772 and R21AI148578 and Washington State University. The content is solely the responsibility of the authors and does not necessarily represent the official views of the National Institute of Allergy and Infectious Diseases, the National Institutes of Health or the United States Department of Agriculture.

## Conflict of Interest

The authors declare that the research was conducted in the absence of any commercial or financial relationships that could be construed as a potential conflict of interest.

## Publisher’s Note

All claims expressed in this article are solely those of the authors and do not necessarily represent those of their affiliated organizations, or those of the publisher, the editors and the reviewers. Any product that may be evaluated in this article, or claim that may be made by its manufacturer, is not guaranteed or endorsed by the publisher.
